# Persistent Pulmonary Hypertension in the Newborn

**DOI:** 10.3390/children4080063

**Published:** 2017-07-28

**Authors:** Bobby Mathew, Satyan Lakshminrusimha

**Affiliations:** Department of Pediatrics, University at Buffalo, Buffalo, NY 14222, USA; slakshmi@buffalo.edu

**Keywords:** oxygen, hypoxemia, nitric oxide, pulmonary blood flow

## Abstract

Persistent pulmonary hypertension of the newborn (PPHN) is a syndrome of failed circulatory adaptation at birth due to delay or impairment in the normal fall in pulmonary vascular resistance (PVR) that occurs following birth. The fetus is in a state of physiological pulmonary hypertension. In utero, the fetus receives oxygenated blood from the placenta through the umbilical vein. At birth, following initiation of respiration, there is a sudden precipitous fall in the PVR and an increase of systemic vascular resistance (SVR) due to the removal of the placenta from circulation. There is dramatic increase in pulmonary blood flow with a decrease in, and later reversal of shunts at the foramen ovale and ductus arteriosus. The failure of this normal physiological pulmonary transition leads to the syndrome of PPHN. PPHN presents with varying degrees of hypoxemic respiratory failure. Survival of infants with PPHN has significantly improved with the use of gentle ventilation, surfactant and inhaled nitric oxide (iNO). PPHN is associated with significant mortality and morbidity among survivors. Newer agents that target different enzymatic pathways in the vascular smooth muscle are in different stages of development and testing. Further research using these agents is likely to further reduce morbidity and mortality associated with PPHN.

## 1. Introduction

During fetal life, pulmonary vascular resistance (PVR) is high and pulmonary blood flow (Qp) is low [1]. Following a normal transition, PVR decreases and Qp increases at birth [2]. Persistent pulmonary hypertension of the newborn (PPHN) is a syndrome of failed circulatory adaptation due to the delay or impairment of the normal fall in PVR that occurs following birth [3]. PPHN is associated with significant mortality and morbidity among survivors. The incidence of PPHN is 1.8 to 2 per 1000 births [4,5] and about 2% in premature infants with respiratory distress syndrome (RDS).

## 2. Pathophysiology of Persistent Pulmonary Hypertension

The fetus is in a state of physiological pulmonary hypertension (PH) [6]. In utero, the fetus receives oxygenated blood from the placenta through the umbilical vein. Oxygenated umbilical venous blood enters the right atrium and is diverted to the left atrium through the foramen ovale. During fetal life, in most mammalian species, the organ of gas exchange (placenta) receives approximately 30–45% of combined ventricular cardiac output [7]. The lungs receive between 8 to 21% [8] of the combined ventricular output through the pulmonary arteries [7]. The pressure gradient drives the flow of blood in the fetal cardiovascular system away from the high resistance low flow pulmonary circulation, towards the low resistance high flow systemic and placental circulation, leading to right to left shunting through the ductus arteriosus into the descending aorta. The high PVR in the fetus is maintained by a combination of anatomical, physical, biochemical and humoral factors. Compression of pulmonary blood vessels by the fluid-filled alveoli, lack of rhythmic distension of the lungs and narrowing of the vascular lumen by the cuboidal configuration of the endothelial cells all play a role in maintaining the elevated PVR in the fetus. Hypoxic pulmonary vasoconstriction is maintained due to the low resting arteriolar and alveolar oxygen tension. Humoral mediators such as endothelin-1, and arachidonic acid metabolites such as leukotrienes and thromboxane and lack of vasodilators such as nitric oxide (NO) and prostacyclin (PGI_2_) contribute to high PVR [2,9]. The low placental systemic vascular resistance in the fetus is maintained by high levels of estrogen, prostaglandins, and NO produced by the placenta.

At birth, following initiation of respiration (increasing alveolar oxygen tension and ventilation) there is a sudden, precipitous fall in the PVR and an increase in SVR due to the removal of the placenta from circulation. Pulmonary endothelial NO acting via the cyclic guanosine monophosphate (cGMP) pathway and PGI_2_ through the cyclic adenosine monophosphate (cAMP) pathway mediate pulmonary vasodilation following birth. There is an eight-fold increase in Qp with a decrease in, and later reversal of shunts at the foramen ovale and ductus arteriosus. The failure of this normal physiological pulmonary transition leads to the syndrome of PPHN often manifesting as hypoxic respiratory failure (HRF).

## 3. Etiology of Persistent Pulmonary Hypertension of the Newborn

**Figure 1:**
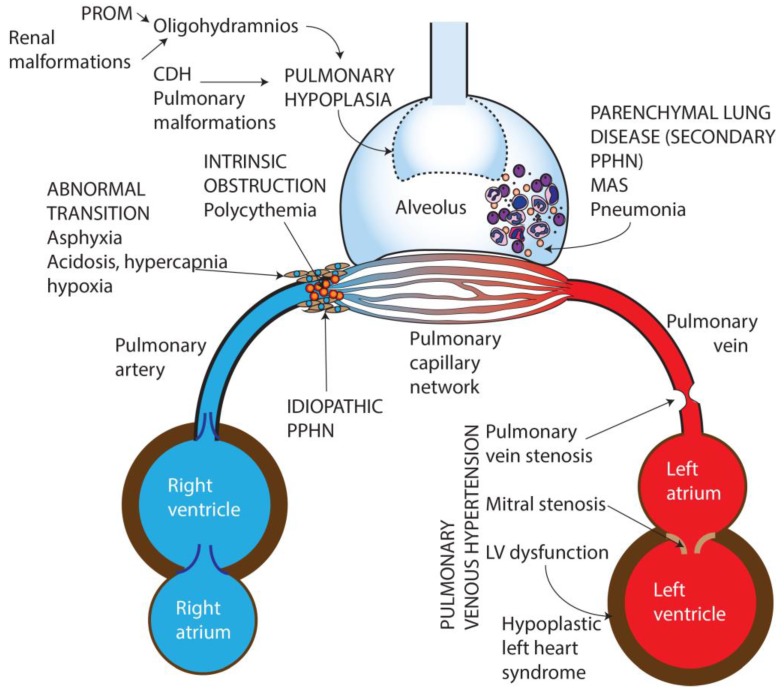
Causes of persistent pulmonary Hypertension in the newborn. PROM—Premature rupture of membranes, CDH—Congenital diaphragmatic hernia, MAS—Meconium aspiration syndrome, PPHN—Persistent pulmonary hypertension of the newborn, LV—Left ventricle. Copyright Satyan Lakshminrusimha.

Based on etiology, PPHN can be categorized into seven broad groups (Figure 1):

1. Idiopathic—No lung disease is present and Qp is decreased due to abnormal vascular remodeling leading to pulmonary vasoconstriction.
2. Abnormal transition at birth—perinatal asphyxia, RDS, and transient tachypnea of newborn, (TTN) resulting in impaired pulmonary vasodilation at birth.
3. Parenchymal disorders (also known as “secondary PPHN”)—such as due to meconium aspiration syndrome (MAS) and pneumonia.
4. Abnormal lung development—pulmonary hypoplasia due to oligohydramnios secondary to renal dysfunction/anomalies or prolonged rupture of membranes, or congenital diaphragmatic hernia (CDH) and other pulmonary malformations.
5. Intravascular obstruction due to hyperviscosity—polycythemia.
6. Pulmonary hypertension (PH) in preterm infants in the initial phase of RDS [10].
7. Pulmonary venous hypertension [11].

### 3.1. Idiopathic PPHN

Idiopathic PPHN is caused by impaired pulmonary vascular relaxation that occurs following birth in the absence of parenchymal lung disease. Histopathological changes include increased vascular smooth muscle proliferation in the pulmonary blood vessels that extend into intra acinar arteries. One of the mechanisms of idiopathic PPHN is due to premature closure of the ductus arteriosus in utero, which forces blood through the constricted pulmonary vasculature leading to increased sheer stress and remodeling. Nonsteroidal anti-inflammatory drugs (NSAIDs) [12] and selective serotonin reuptake inhibitors (SSRIs) taken during pregnancy [13] have been associated with PPHN in the offspring but more recent studies have reported conflicting results [14]. Decreased endogenous NO synthesis (as can be associated with urea cycle enzyme defects) [15], bioavailability of NO, and vascular responsiveness to vasodilators may play a role in the etiopathogenesis of this condition. This condition is also called black lung PPHN due to the characteristic appearance on chest X-ray due to lack of lung disease and decreased pulmonary vascularity.

### 3.2. Abnormal Pulmonary Transition

Abnormal pulmonary transition at birth, due to for example perinatal asphyxia can lead to hypoxemia, hypercarbia and metabolic acidosis, all of which cause pulmonary vasoconstriction and increased intra- and extra-pulmonary shunting. Decreased alveolar recruitment and lung volume can cause physical constriction of the intrapulmonary blood vessels further impeding pulmonary blood flow. Optimizing lung recruitment to functional residual capacity (FRC) and effective ventilation often lead to reversal of the pathophysiological process associated with these conditions. Preterm infants with severe RDS and infants born by cesarean section without labor experience a slower decrease in PVR compared to term infants born by vaginal delivery.

### 3.3. Parenchymal Lung Disease

Sepsis, pneumonia and MAS cause parenchymal lung disease with variable degrees of chemical pneumonitis, surfactant inactivation and release of proinflammatory mediators that increase levels of vasoconstrictors such as endothelin and thromboxane. Ventilation–perfusion (V/Q) mismatch associated with these conditions causes hypoxemia, worsening pulmonary vasoconstriction. The decrease in SVR associated with sepsis, combined with exacerbation of PVR due to hypoxemia, increase the right to left shunt at the ductus arteriosus and foramen ovale.

### 3.4. Pulmonary Hypoplasia

Pulmonary hypoplasia secondary to CDH, thoracic dystrophy or prolonged oligohydramnios (from renal dysplasia or preterm premature rupture of membranes) leads to impaired parenchymal and pulmonary vascular development. In addition, CDH is often associated with abnormalities of cardiac development and function. Left ventricular dysfunction and pulmonary venous hypertension frequently complicate the clinical course in infants with CDH [16,17].

### 3.5. Prematurity

Extremely premature infants are at risk of PH/HRF in the first few days of postnatal life. The fetal PVR is high during the canalicular stage secondary to paucity of pulmonary vascular network reducing the cross sectional area of the immature pulmonary vascular bed. Pulmonary vasculature at mid-gestation is less responsive to oxygen mediated pulmonary vasodilation. These infants typically respond poorly to inhaled pulmonary vasodilators such as iNO [18]. However, there is a subset of preterm infants with prolonged rupture of membranes, and oligohydramnios with typical term-PPHN physiology who may have a better response to iNO [19].

### 3.6. Pulmonary Venous Hypertension

These conditions present with HRF and can be clinically indistinguishable from pulmonary arterial hypertension [20]. The chest X-ray appearance shows increased pulmonary vascularity and fluid congestion. The decrease in pulmonary blood flow is due to backpressure from impaired venous drainage into the left atrium (Figure 1). Left-sided obstructive congenital heart disease (mitral atresia, hypoplastic left heart syndrome, critical coarctation of aorta), total anomalous pulmonary venous connection with obstruction [21], pulmonary vein stenosis can all present with varying degrees of pulmonary venous hypertension. Left ventricular dysfunction due to asphyxia, sepsis or CDH can also present with pulmonary venous hypertension. The diagnosis is made on echocardiography by evaluating the direction of fetal shunts. Inhaled NO is contraindicated in these conditions as it increases pulmonary edema and causes deterioration of gas exchange [22].

## 4. Clinical Presentation

PPHN presents with varying degrees of HRF. (For the differential diagnosis of hypoxemia in neonates—see Table 1) The characteristic findings of PPHN are labile hypoxemia and pre—post ductal saturation gradient (manifested clinically as differential cyanosis). Infants with PPHN have wide swings in arterial oxygen saturation levels due to acute changes in pulmonary blood flow and right-to-left shunt associated with episodic changes in PVR in response to minimal stimulation. A pre to post ductal gradient of greater than 10% is common in infants with PPHN [23] Physical examination findings are often minimal with a loud second heart sound and systolic murmur of tricuspid regurgitation. A chest X-ray is often helpful to diagnose the underlying cause and to assess response to changes with ventilator therapy.

### 4.1. Echocardiography

**Figure 2:**
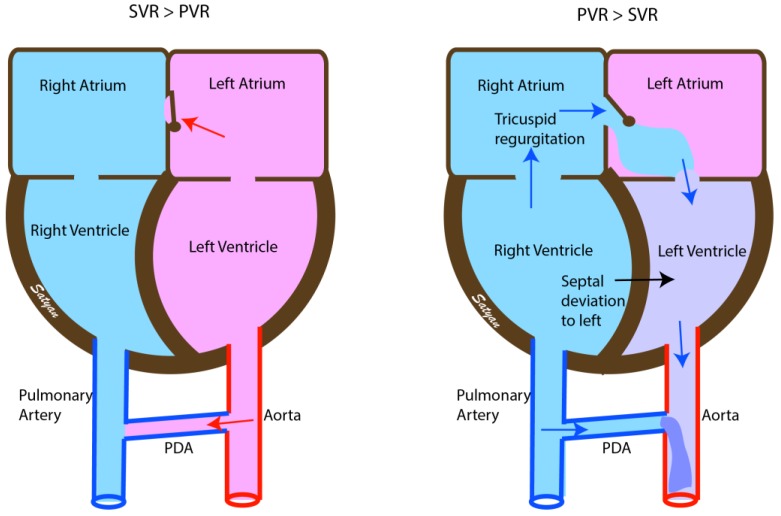
Echocardiographic findings in normal infants (**left**) and in PPHN (**right**). Soon after birth the pressures within the left-sided chambers of the heart are higher than in the right and the fetal shunts are reversed. The interatrial shunt and the shunt across the patent ductus arteriosus (PDA) is left to right. In infants with PPHN the pressures remain elevated in the right atrium and ventricle with right to left shunt at the atrial level and at the PDA causing desaturation (due to interatrial shunt) and differential cyanosis (due to PDA). There is right ventricular hypertrophy with bulging of the interventricular septum to the left and tricuspid regurgitation. SVR—Systemic vascular resistance, PVR—Pulmonary vascular resistance. Copyright Satyan Lakshminrusimha.

Right ventricular hypertrophy, deviation of the interventricular septum to the left, tricuspid regurgitation (TR) and right to left or bidirectional shunting at the patent foramen ovale (PFO) and patent ductus arteriosus (PDA) are the cardinal echocardiographic findings in PPHN (Figure 2). The right ventricular systolic pressure can be estimated using the modified Bernoulli equation as 4v^2^ + right atrial pressure, where v = maximal velocity of the TR jet in m/s. Changes in pulmonary arterial pressure, myocardial function and blood flow patterns across the ductus and foramen ovale can be monitored over time by echocardiography. Echocardiograms can assess response to therapy in infants with PPHN [24].

The direction of blood flow across the fetal shunts PDA and PFO provide critical information about the diagnosis and response to interventions in infants with HRF.

In infants with hypoxemia and left-to-right shunt at both PDA and PFO, the hypoxemia is due to intrapulmonary shunting and interventions to improve lung recruitment such as optimizing lung volume with positive end expiratory pressure (PEEP) and providing adequate mean airway pressure and or surfactant should be attempted.

Right-to-left shunt at both PDA and PFO is suggestive of increased PVR and extrapulmonary shunting and is likely due to PPHN. Inhaled pulmonary vasodilator therapy such as iNO should be the primary therapy after optimizing lung recruitment in these patients.

Right-to-left shunt at the ductus arteriosus and left-to-right shunt at the PFO is suggestive of pulmonary hypertension with left ventricular dysfunction and is often seen in CDH, asphyxia and sepsis. In this setting, pulmonary vasodilator therapy alone may worsen oxygenation by causing increasing fluid congestion in the lungs. Pulmonary vasodilator therapy combined with inotropy with milrinone may be helpful in this situation by supporting left ventricular function. A similar shunting pattern is also seen in infants with pulmonary vein stenosis, and congenital heart disease with ductal dependent systemic blood flow as in hypoplastic left heart syndrome, critical aortic stenosis, mitral atresia, interrupted aortic arch, and critical coarctation of aorta.

Left-to-right shunt at the ductus arteriosus and right-to-left shunt at the foramen ovale is seen in infants with cyanotic congenital heart disease with ductal dependent pulmonary blood flow such as tricuspid atresia, critical pulmonary stenosis and pulmonary atresia. It is important to exclude congenital heart disease prior to initiation of pulmonary vasodilator therapy in infants presenting with HRF.

## 5. Assessment of Severity of HRF and Monitoring Response to Therapy

Objective assessment of oxygenation in infants with PPHN/HRF can be performed with one of the following indices:

- Oxygenation index (OI) = FiO_2_ × mean airway pressure × 100/PaO_2_
  Severity of HRF based on OI:
  ○ Mild ≤15
  ○ Moderate 15 to ≤25
  ○ Severe 25 to ≤40 and
  ○ Very severe >40
- Alveolar—arterial oxygen gradient (A-a gradient or A-aDO_2_). This estimates the partial pressure gradient of oxygen from the alveolus to the aorta.
  A-aDO_2_ = [FiO_2_ × (Barometric pressure − water vapor pressure) − PaCO_2_/R)] − PaO_2_.
  ○ Where R is the respiratory quotient (R = 1, in an infant receiving exclusive intravenous dextrose and approximately 0.8 when on mixed diet).
  ○ The normal A-aDO_2_ is 4–20 mm of Hg. A-aDO_2_ can be above 600 mm Hg in very severe cases of HRF. An online calculator is available at http://perinatology.com/calculators/A-a%20gradient.htm.
- P/F ratio is the ratio of the partial pressure of oxygen (in mm Hg) in the arterial blood to the fractional inspired oxygen concentration. P/F ratio = PaO_2_/ FiO_2_
  Severity assessment based on P/F ratio:
  ○ Mild >200 to ≤300.
  ○ Moderate >100 to ≤200 and
  ○ Severe ≤100 mm Hg.
- Note: For calculation of OI and P/F ratios, it is preferable to use preductal blood gases [25]. Preductal PaO_2_ accurately predicts oxygen delivery to vital organs such as the brain and heart and is not altered by right to left shunting at the PDA. However, many patients with PPHN have umbilical arterial access (postductal blood gases) resulting in lower PaO_2_ and higher OI and lower P/F ratio compared to preductal evaluation.
- Oxygen saturation index (OSI)—All of the above indices require arterial blood gas for evaluation and hence the need for arterial access. OSI is a noninvasive index of gas exchange and is calculated as follows:
  ○ OSI = Mean airway pressure × FiO_2_ × 100/Preductal SpO_2_.
  ○ OSI has been shown to correlate well in infants with HRF, OI ≈ 2 × OSI [26].

## 6. Management

The goals of therapy in PPHN are to promote lung recruitment, pulmonary vasodilation, improve oxygenation and provide adequate oxygen delivery to the tissues while minimizing oxidative stress and free radical injury.

### 6.1. Supportive Therapies

Maintaining normal body temperature, glucose level, ionic calcium and sedation/ analgesia are important for optimal outcomes in patients with PPHN.

### 6.2. Lung Recruitment

A common cause of failure to respond to treatment in HRF with impaired oxygenation and ventilation is inadequate lung recruitment. Optimizing lung recruitment with the use of PEEP to achieve lung expansion to functional residual capacity (FRC) approximately 8–9 ribs expansion on anteroposterior chest X-ray) often ensures adequate lung recruitment. Underinflation and overinflation increase PVR due to the mechanical effects on the extra-alveolar and intra-alveolar pulmonary blood vessels. Atelectasis increases intrapulmonary right to left shunting leading to worsening hypoxia and hypercarbia. Overinflation can impede venous return and cause systemic hypotension.

### 6.3. Oxygenation

Oxygen is a potent vasodilator and hypoxia causes pulmonary vasoconstriction. Supplemental oxygen should be provided to achieve normoxia (PaO_2_ between 50 and 80 mmHg) [27]. Hyperoxia (PaO_2_ > 100 mmHg) does not enhance pulmonary vasodilation [28,29] and increases the formation of oxygen free radicals that increases pulmonary arterial contractility [30,31] and impairs vasodilator response to iNO. The optimal saturation target range for patients with PPHN is not known. Targeting a lower limit of preductal SpO_2_ of 92% provides a buffer to hypoxic pulmonary vasoconstriction and an upper limit of 97% ensures the optimal balance of pulmonary vasodilation and minimizes adverse effects from oxidative stress [32,33]. Adequacy of tissue oxygen delivery should be followed closely by pulse oximetry and blood gas monitoring for acidosis. Near infrared spectroscopy (NIRS) is a non-invasive assessment of oxygen delivery and is commonly used in postoperative management of congenital heart disease. Although not routinely used in the management of PPHN, there may be benefit to the use of NIRS in the presence of PPHN associated with hypoxic ischemic encephalopathy.

### 6.4. Surfactant Replacement Therapy

Surfactant inactivation occurs with MAS, pneumonia and sepsis. Surfactant therapy leads to improvement in oxygenation by improving V/Q matching and decreases intrapulmonary shunting. In newborn infants with parenchymal lung disease, surfactant replacement prior to initiation of iNO improves outcome and reduces the need for extracorporeal membrane oxygenation (ECMO) [34].

### 6.5. Inhaled Nitric Oxide (iNO)

**Figure 3:**
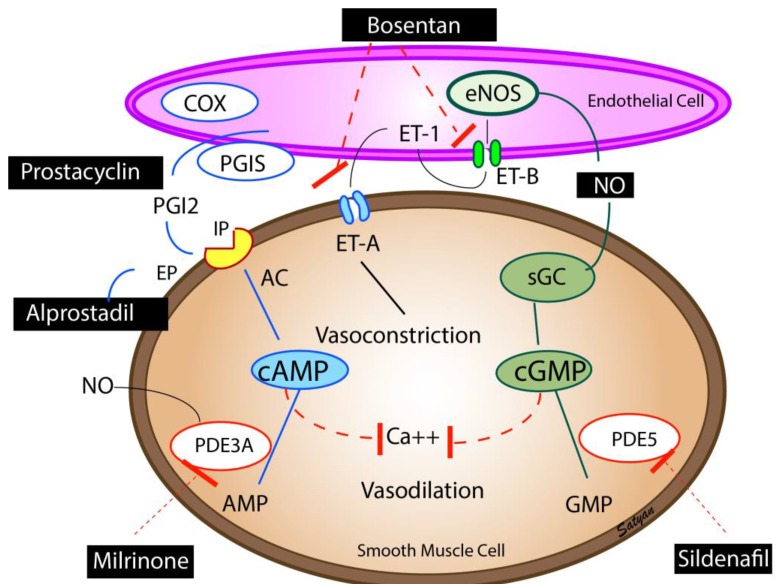
Pulmonary vasodilators—Endothelium-derived vasodilators: prostacyclin (PGI_2_), nitric oxide (NO), and vasoconstrictor (endothelin, ET-1). The enzymes, cyclooxygenase (COX) and prostacyclin synthase (PGIS) are involved in the synthesis of prostacyclin. Prostacyclin through (PGI_2_ receptor (IP) stimulates adenylate cyclase (AC) to produce cAMP. cAMP is broken down by phosphodiesterase 3A (PDE3A) in the smooth muscle cell. Milrinone inhibits PDE 3A and increases cAMP levels in pulmonary arterial smooth muscle cells and cardiac myocytes resulting in vasodilation and inotropy. Endothelin is a powerful vasoconstrictor and acts on ET-A receptors in the smooth muscle cell and increases ionic calcium concentration. A second endothelin receptor (ET-B) on the endothelial cell stimulates NO release and vasodilation. Endothelial nitric oxide synthase (eNOS) catalyzes the production of NO which diffuses from the endothelium to the smooth muscle cell and stimulates soluble guanylate cyclase (sGC) enzyme to produce cyclic guanosine monophosphate (cGMP). cGMP is broken down by the PDE5 enzyme in the smooth muscle cell. Sildenafil inhibits PDE5 and increases cGMP levels in pulmonary arterial smooth muscle cells. cAMP and cGMP reduce cytosolic ionic calcium concentrations and induce smooth muscle relaxation and pulmonary vasodilation. NO is a free radical and avidly combines with superoxide anions to form a toxic vasoconstrictor, peroxynitrite. The bioavailability of NO in a tissue is determined by the local concentration of superoxide anions. Hyperoxic ventilation can increase the risk of formation of superoxide anions in the pulmonary arterial smooth muscle cells and limit the bioavailability of NO. Copyright Satyan Lakshminrusimha.

Inhaled nitric oxide remains the only United States Food and Drug Administration (FDA)-approved pulmonary vasodilator for infants with PPHN. Nitric oxide is produced by the endothelial cells and causes pulmonary vasodilation through generation of cyclic guanosine monophosphate (cGMP) (Figure 3). Inhaled NO causes selective vasodilation of the pulmonary circulation as it diffuses from the alveolus into the smooth muscle cells and causes pulmonary vasodilation [35]. Inhaled NO is inactivated by hemoglobin in the circulation and hence has minimal systemic vasodilator effect. Inhaled NO causes vasodilation of pulmonary blood vessels that are adjacent to well-ventilated alveoli and decreases intrapulmonary right-to-left shunting and improves V/Q matching (microselective effect).

**Figure 4:**
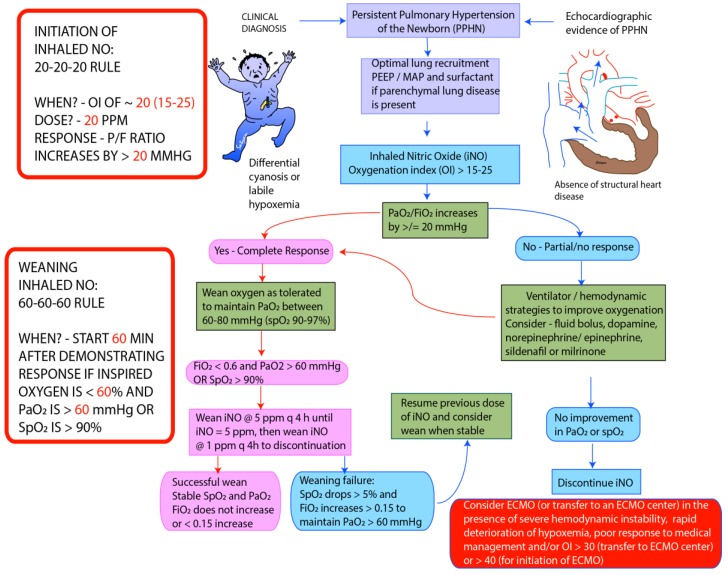
Guidelines for initiation and weaning iNO in PPHN/hypoxic respiratory failure (HRF) in Neonatal Intensive Care Unit (NICU) (adapted from the protocol at Women & Children’s Hospital of Buffalo). The recommended starting dose of iNO is 20 parts per million (ppm). Improvement in PaO_2_ ≥ 20 mm Hg or hemoglobin saturation by pulse oximetry ≥ 5% is considered complete response. In patients who fail to respond iNO, measures needed to optimize of lung recruitment and hemodynamics need to be undertaken. iNO should be discontinued if there no response. In responders wean FiO_2_ initially while maintaining PaO_2_ between 60 and 80 mm Hg. Once PaO_2_ is stable and FiO_2_ is below 0.6, start weaning iNO by 5 ppm every 4 h till 5 ppm. Below 5 ppm wean iNO by 1 ppm every 4 h. During weaning >5% drop in pulse oximetry or sustained increase in FiO_2_ > 0.15 to maintain PaO_2_ > 60 mm Hg is considered weaning failure, and previous dose of iNO should be resumed. Weaning should be resumed once stable. Monitor methemoglobin levels at baseline, 2 and 8 h following initiation and every 48 h thereafter [37]. PEEP—Positive End Expiratory Pressure, MAP—Mean Airway Pressure, ECMO—Extracorporeal Membrane Oxygenation. Copyright Satyan Lakshminrusimha.

Inhaled NO is usually initiated when the oxygenation index reaches 15–25. Delay in initiation of pulmonary vasodilator therapy has been shown to be associated with worsening of HRF. The starting dose of iNO is usually 20 ppm. Higher doses do not enhance pulmonary vasodilation and may increase the incidence of side effects. Clinical response is defined as an improvement in PaO_2_ by at least 20 mm Hg following initiation of iNO. In infants who fail to respond to iNO after optimizing lung recruitment, ventilation and hemodynamic support, it is important to discontinue iNO to prevent downregulation of endogenous NO pathway [36] and prevent injury by formation of peroxynitrite [32]. Once oxygenation response is achieved, iNO dose can be weaned in decrements of 5 ppm till 5 ppm is reached and by 1 ppm subsequently (see weaning protocol) [37]. It is important to monitor methemoglobin and nitrogen dioxide levels at initiation and daily during treatment with iNO. The approach to weaning iNO used in our center is shown in Figure 4.

### 6.6. Prostaglandins

Prostacyclins are the mainstay of therapy for pulmonary hypertension in adults. Evidence for use in newborns with PPHN is limited to case series. Prostaglandins cause activation of adenylate cyclase increasing the cAMP levels in the vascular smooth muscle cells leading to pulmonary vasodilation (Figure 3) [38]. Intravenous prostacyclins can lead to systemic hypotension and worsening of VQ mismatch. Inhaled PGI_2_ by nebulization has been shown to be effective in infants who had poor response to iNO [39].

### 6.7. Phosphodiesterase Inhibitors

Cyclic mononucleotides cGMP and cAMP are degraded by phosphodiesterase (PDE) enzymes in the pulmonary artery smooth muscle cells. Milrinone is a PDE3A inhibitor and enhances cAMP levels in the arterial smooth muscle cells and cardiac myocytes resulting in vasodilation and inotropy (Figure 3). Milrinone is administered as a loading dose of 50 µg/kg/min given over 30 min followed by 0.33 µg/kg/min as a continuous infusion. The dose may be titrated up to 1 µg/kg/min with close monitoring of systemic blood pressure.

Sildenafil is a PDE5 inhibitor, which decreases breakdown of cGMP resulting in pulmonary vasodilation (Figure 3) [40]. Sildenafil may be used in combination with iNO, or may be used alone to prevent rebound PH following withdrawal of iNO. Hyperoxic ventilation increases PDE5 [41], and PDE5 inhibitors may be particularly effective in patients who have been exposed to hyperoxic ventilation. The typical dose of intravenous sildenafil is a loading dose of 0.42 mg/kg over 3 h followed by 0.07 mg/kg/h [42]. The starting dose of oral sildenafil is 0.5 mg/kg/dose every eight h and can be increased to a maximum of 3–8 mg/kg/day, divided every 6–8 h. Phosphodiesterase inhibitors dilate the pulmonary vasculature irrespective of ventilation status and could cause V/Q mismatch leading to worsening oxygenation. Systemic vasodilation and hypotension are particularly common during initiation of therapy, hence intravascular volume status should be optimized, and blood pressure monitored closely during treatment with phosphodiesterase inhibitors.

### 6.8. Inotropes

Systemic hypotension due to low systemic vascular resistance or left ventricular dysfunction is common in infants with PPHN. Right ventricular dysfunction and failure occurs due to increased afterload in infants with severe PPHN. Optimizing cardiac output with adequate volume expansion and inotropic support is important for achieving optimal gas exchange and systemic oxygen delivery.

(1) In the presence of systemic hypotension without cardiac dysfunction, the agents of choice are dopamine, norepinephrine and vasopressin (pressor support).
(2) When systemic hypotension is associated with cardiac dysfunction, epinephrine or a combination of dopamine/vasopressin and milrinone are the agents of choice.
(3) In the presence of stable systemic blood pressure and cardiac dysfunction, milrinone is the agent of choice.
(4) Studies in the lamb model of PPHN have shown increase in pulmonary arterial pressure and decreased pulmonary blood flow following initiation of dopamine. In control lambs with normal pulmonary vasculature, dopamine increases the systemic blood pressure with relatively small increase in pulmonary arterial pressure. In contrast, lambs with remodeled pulmonary vasculature and PPHN induced by antenatal ductal ligation, dopamine, especially at doses >10 µg/kg/min, results in a greater increase in pulmonary arterial pressure [9].
(5) The use of dobutamine is often associated with a fall in systemic blood pressure resulting in exacerbation of right to left shunting and systemic desaturation. Dobutamine causes increase in myocardial oxygen requirement which may worsen myocardial dysfunction in PPHN.

Maintaining adequate (physiological) systemic blood pressure decreases systemic oxygen desaturation by decreasing the pulmonary to systemic shunt through the ductus arteriosus. It is a common practice to increase systemic blood pressure to supraphysiological levels above pulmonary arterial pressures to prevent right-to-left shunting and desaturation [43]. Iatrogenic systemic hypertension does not improve oxygen delivery to the brain or myocardium (preductal), but may increase stress on an already failing right ventricle. The right-to-left shunt at PDA acts as a pop off against high right ventricular afterload, and increases shear stress on the pulmonary arterial endothelium. In addition, the vasopressor effect of commonly used inotropic agents is not selective to systemic vasculature and may increase the pulmonary arterial pressure as well [9].

### 6.9. Sedation/Paralysis

In PPHN, episodes of pulmonary vasoconstriction can be induced by external stimuli. Hence, these infants should be nursed in an environment with minimal stimulation and provided adequate sedation with fentanyl/morphine. The routine use of paralyzing agents should be avoided [4] and reserved for infants who need high ventilator settings to maintain oxygenation and ventilation in the target range.

### 6.10. Nutrition

The goal is to provide adequate parenteral calories and maintaining adequate ionic calcium levels for optimal cardiac function by providing a parenteral nutrition solution with glucose, calcium, amino acids and lipids.

### 6.11. Acid–Base Balance

Acidosis promotes pulmonary vasoconstriction and hence it is important to maintain pH in the normal range (7.25–7.4). Metabolic alkalosis by sodium bicarbonate infusion and respiratory alkalosis by hyperventilation should be avoided [4] as it has been shown to increase the risk of developmental delay and sensorineural deafness [44]. PaCO_2_ should be maintained between 40 and 55 mm Hg.

### 6.12. Extracorporeal Membrane Oxygenation (ECMO)

About 30–40% of infants do not achieve a sustained oxygenation response to iNO. ECMO may be lifesaving in infants who fail maximal conventional therapy. Hence, access to ECMO or ability to transfer patients in a timely manner to an ECMO center should be available to centers that manage patients with HRF/PPHN. The indications for ECMO are OI >40 for >2 h in 3 out of 5 arterial blood gases or A-aDO_2_ ≥ 630 for >4 h. Hypoxemia to this degree is predictive of 60–80% mortality in the absence of access to ECMO. The risk of rapid deterioration and death is high in patients with severe PPHN and HRF and hence discussions and initiation of transfer should be undertaken in patients at an OI of 30 on pulmonary vasodilator therapy. Contraindications to ECMO include gestational age <34 weeks (due to high risk of intraventricular hemorrhage (IVH)), significant IVH, persistent bleeding diathesis, irreversible neurological injury, major chromosomal abnormalities and irreversible major organ dysfunction.

## 7. Follow-Up

Follow-up studies comparing therapies for PPHN demonstrate that neurodevelopmental impairments are present in about 25% of infants, sensorineural hearing loss in 6–23% and persisting respiratory illness in 25%. It is important to ensure long-term multidisciplinary follow up care following discharge for these infants.

## 8. Conclusions

Survival of infants with PPHN has significantly improved with the use of gentle ventilation, surfactant and iNO. However, due to the limited availability of iNO, high cost, and lack of efficacy in about a third of patients with PPHN, there is a need to develop better agents that target different enzymatic pathways in the vascular smooth muscle. Newer agents such as l-Citrulline, endothelin receptor antagonists, soluble guanylyl cyclase stimulators and activators, Rho kinase inhibitors, peroxisome proliferator activated receptor gamma (PPAR ɤ) agonists, and antioxidants are in different stages of development and testing [45]. Further research using these newer agents is likely to reduce morbidity and mortality associated with PPHN.

## Figures and Tables

**Table 1:** Differential diagnosis of hypoxemia in newborn infants.

	Lung Disease without PPHN	Cyanotic Congenital Heart Disease	PPHN
History	Fetal distress, PROM, chorioamnionitis	Antenatal diagnosis	Often negative other than in secondary PPHN
Respiratory distress	Present	Usually absent	Often present
Oxygen saturation on pulse oximetry	Improves with supplemental oxygen	Fixed low saturations Minimal response to supplemental oxygen	Labile saturations. Differential cyanosis
Hyperoxia test *	PaO_2_ often > 150 mm Hg	PaO_2_ often < 100 mm Hg	PaO_2_ often > 100 mm Hg
PaCO_2_	Elevated	Normal/low	Often elevated (except in idiopathic PPHN)
Hyperoxia-Hyperventilation *	PaO_2_ > 150 mm Hg	PaO_2_ often < 100 mm Hg	PaO_2_ improves with hyperventilation
Chest X ray	Abnormal	Abnormalities of cardiac silhouette and pulmonary vascularity	Decreased vascularity in idiopathic PPHN
Echocardiogram	Normal	Structural cardiac abnormalities	Structurally normal heart (see text for characteristic echo findings of PPHN)

* Both hyperoxia and hyperoxia hyperventilation tests are not used routinely in clinical practice due to increased availability of echocardiography in most institutions. These tests expose the infant to hyperoxia and hypocarbia with the potential to cause oxidative stress and decreased cerebral perfusion respectively.
